# Robotic-assisted spine surgery allows for increased pedicle screw sizes while still improving safety as indicated by elevated triggered electromyographic thresholds

**DOI:** 10.1007/s11701-022-01493-8

**Published:** 2022-11-30

**Authors:** Charles W. Kanaly, Danielle M. Backes, Nader Toossi, Brandon Bucklen

**Affiliations:** 1 Steward St. Anne’s Hospital, Fall River, MA USA; 2 Neurosurgery Center of Southern New England, PC, Fall River, MA USA; 3 Musculoskeletal and Education Research Center, Audubon, PA USA

**Keywords:** Robotic-assisted spine surgery, Triggered EMG stimulation, Posterior spinal fixation

## Abstract

The present study used triggered electromyographic (EMG) testing as a tool to determine the safety of pedicle screw placement. In this Institutional Review Board exempt review, data from 151 consecutive patients (100 robotic; 51 non-robotic) who had undergone instrumented spinal fusion surgery of the thoracic, lumbar, or sacral regions were analyzed. The sizes of implanted pedicle screws and EMG threshold data were compared between screws that were placed immediately before and after adoption of the robotic technique. The robotic group had significantly larger screws inserted that were wider (7 ± 0.7 vs 6.5 ± 0.3 mm; *p* < 0.001) and longer (47.8 ± 6.4 vs 45.7 ± 4.3 mm; *p* < 0.001). The robotic group also had significantly higher stimulation thresholds (34.0 ± 11.9 vs 30.2 ± 9.8 mA; *p* = 0.002) of the inserted screws. The robotic group stayed in the hospital postoperatively for fewer days (2.3 ± 1.2 vs 2.9 ± 2 days; *p* = 0.04), but had longer surgery times (174 ± 37.8 vs 146 ± 41.5 min; *p* < 0.001). This study demonstrated that the use of navigated, robot-assisted surgery allowed for placement of larger pedicle screws without compromising safety, as determined by pedicle screw stimulation thresholds. Future studies should investigate whether these effects become even stronger in a later cohort after surgeons have more experience with the robotic technique. It should also be evaluated whether the larger screw sizes allowed by the robotic technology actually translate into improved long-term clinical outcomes.

## Introduction

Pedicle screw fixation in spinal surgery is a widely accepted procedure to correct spinal alignment, provide stabilization following neural decompression, and allow fusion to occur [1–6]. Pedicle screw placement in the lumbar and lower thoracic areas provides spinal fixation while decreasing the risk of postoperative complications [2, 7]. However, the pedicle is surrounded by many sensitive biological entities such as the nerve roots, and these are usually not completely visualized during pedicle screw insertion. Pedicle screw misplacement may therefore lead to peripheral nerve injury in the lumbosacral spine [7, 8]. This occurs in approximately 4.2% of patients, and nerve injury may lead to serious postoperative motor and sensory deficits along with patient immobility, discomfort, and pain [7–9].

Computer-assisted navigation and surgical robotics are becoming increasingly common because they offer the abilities to direct and confirm pedicle screw placement with increased accuracy. These technologies also may decrease radiation exposure to the surgical staff, and decrease surgery and recovery time [10–12].

**Fig. 1:**
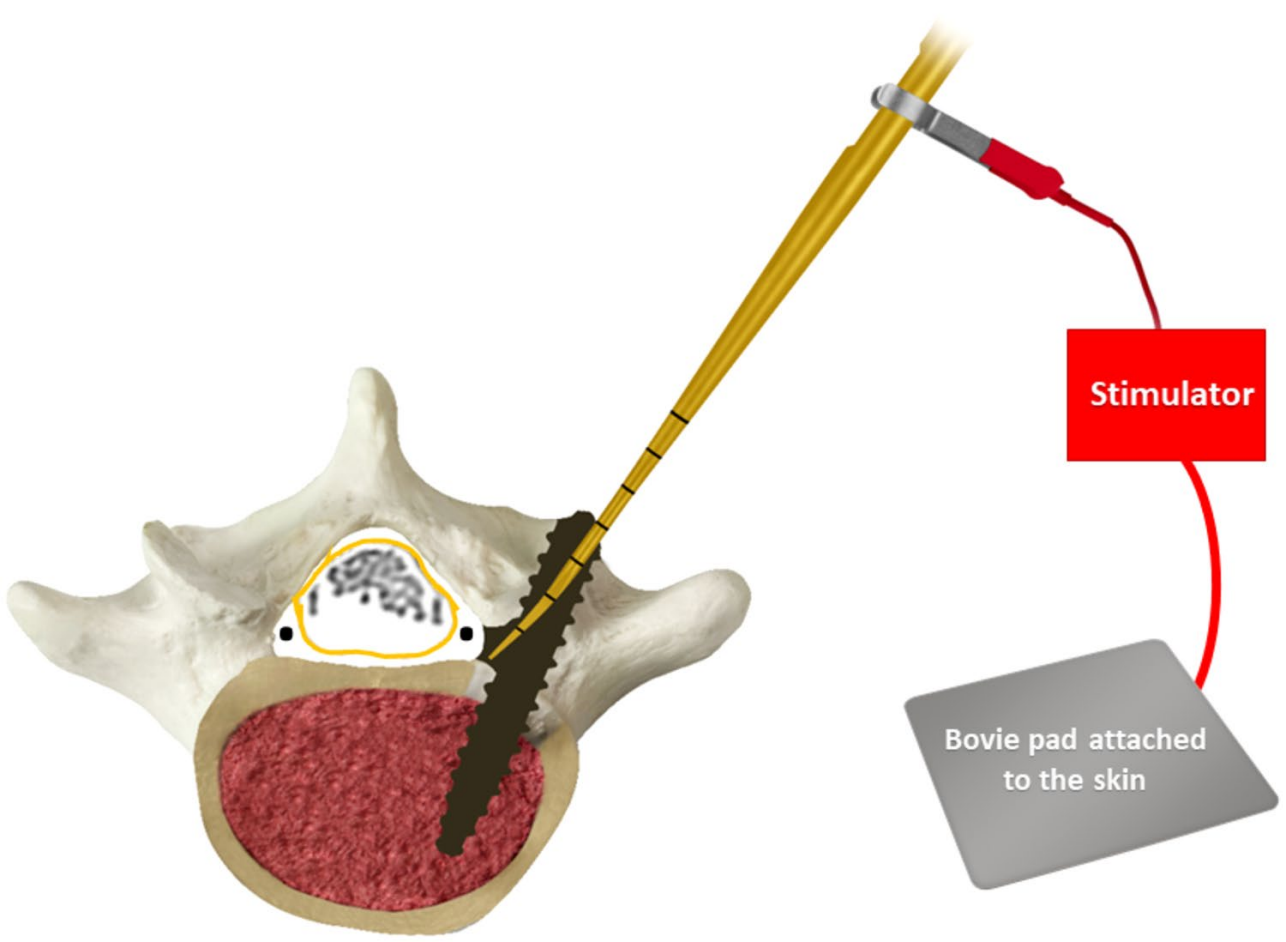
Stimulation of a traversing nerve root in the spinal canal by the probe through a medial pedicle wall breach. The muscles innervated by the nerve root will have an evoked potential picked up by electromyogram

Triggered electromyograph(ic) (EMG) or pedicle stimulation is a technique that involves the electrical stimulation of a pedicle opening or screw to evoke muscle action potential and subsequent contractions of muscle fibers that can be recorded by carefully placed electrodes [13]. The number of milliamps required to evoke a muscle response can be measured as a threshold value, and this value will differ depending on the proximity of the stimulation source to the nerve root, as well as the conductivity of the intervening tissue. High stimulation thresholds indicate a safe screw placement due to increased resistance to current flow, whereas low stimulation thresholds are indicative of a pedicle screw breach, or proximity to an exiting or traversing nerve root [14–16]. Studies have shown that the use of EMG monitoring may increase the safety of pedicle screw placement by detecting pedicle wall breaches, and may thereby minimize the risk of patient injury [14, 17] (Fig. 1).

Although previous studies in the literature have investigated the accuracy of screws placed with the assistance of robots [18–20], studies exploring the safety of robotic-assisted screw placement are limited. To the authors’ knowledge, this is the first study to compare EMG stimulation threshold (STIM) data of patients who underwent spinal fusion surgery with robot-navigated assistance to that of patients who had spinal fusion surgery without the use of robotic technology. The purpose of this study is to retrospectively compare the sizes of inserted pedicle screws and STIM measurements between robotic versus non-robotic cohorts.

## Materials and methods

A retrospective institutional review board-exempt study was conducted of 151 consecutive patients who underwent instrumented spinal fusion surgery of the thoracic, lumbar, or sacral regions at a single facility. The first 100 patients who underwent surgery at this location with navigated, robotic assistance utilizing the ExcelsiusGPS^®^ robot (Globus Medical, Inc., Audubon, PA, USA) were compared to the 51 patients who underwent free-hand/fluoroscopically guided surgery immediately prior to adoption of this robotic technique. All of the surgeries occurred between May 2017 and December 2018. The robotic technique was implemented in December 2017, and so all surgeries in the study after that point were performed with robotic assistance. All adult patients who had undergone spine surgery with posterior stabilization were included in the study to encompass the first 100 robotic patients and as far back as possible prior to robot adoption for which triggered EMG threshold values were able to be obtained. A midline open surgical technique was performed in all 51 non-robotic and in 69 of the 100 robotic surgeries, while the remaining surgeries used a Wiltse paraspinal minimally invasive approach [21]. Data were collected retrospectively from patient records without any identifying information. Patient demographics, intraoperative, and EMG threshold data were analyzed. STIM measurements were obtained by placing the stimulation probe on the shaft of the screw after the screw had been inserted. STIM measurements that were recorded intraoperatively as a range were entered in the final analysis as the lowest value in the range.

### Navigated, robot-assisted pedicle screw positioning system

All screws in the robotic group were inserted utilizing the robot positioning system (Excelsius GPS^®^; Globus Medical, Inc. Audubon, PA, USA). This system uses real-time surgical navigation and robotic direction in conjunction with a dynamic reference base and positioning camera to guide pedicle screw placement. This system allows for preoperative planning, followed by robotic-assisted intraoperative implant placement with navigated instruments.

### Statistical analysis

Data were analyzed using SPSS v20.0.0 software for Windows (IBM Corp., Armonk, New York). Demographic data such as age and body mass index (BMI) were reported as averages, while gender and diagnosis were reported as frequencies. Intraoperative data such as surgical time, radiation time, blood loss, and length of stay were reported as averages, while treatment type, surgical level and intraoperative complications were reported as frequencies. Parametric and nonparametric tests were used to assess differences between groups. Statistical significance level was set at 0.05.

## Results

The most common indications for fusion surgery were degenerative spondylolisthesis and stenosis (64%) followed by iatrogenic instability (16%).

Demographic characteristics were similar between the robotic and non-robotic groups (Table 1). Fifty-four percent of patients in the robotic and 55% in the non-robotic group were female. The mean patient ages were 63 and 64 for the robotic and non-robotic groups, respectively. The mean patient BMIs were 31.2 and 29.7 kg/m^2^ for the robotic and non-robotic groups, respectively.

**Table 1:** Patient demographics

	Robot	No robot	*p* value
Number of patients	100	51	
Gender			
Female, *n* (%)	54 (54%)	28 (55%)	
Male, *n* (%)	46 (46%)	23 (45%)	
Age (mean ± SD)	63 ± 11.2	64 ± 12.2	0.64
BMI (mean ± SD)	31 ± 6.3	30 ± 6.7	0.19
Mean number of screws/case	4.5	4.7	0.83
Mean number of vertebrae/case	2.3	2.4	0.77

*n* number, *BMI* Body mass index, *SD* standard deviation

Operative data and differences between groups can be found in Table 2. No significant differences between groups were observed for fluoroscopy time or blood loss. Postoperative hospital stays for the robotic group were significantly shorter than for the non-robotic group (2.3 ± 1.2 vs 2.9 ± 1.9 days; *p* = 0.04), while the non-robotic group had significantly shorter operative times (174 ± 37.8 vs 146 ± 41.5 min; *p* < 0.001). The robotic group had a mean of 4.5 screws and 2.3 vertebrae per patient, whereas the non-robotic group had a mean of 4.7 screws and 2.4 vertebrae per patient.

**Table 2:** Surgical data

	Robot	Non-robotic	*p* value
Operative time (minutes)	174 ± 37.8	146 ± 41.5	< 0.001*
Fluoroscopy time (seconds)	14 ± 6.5	13.4 ± 7.1	0.66
Blood loss (cc)	129.1 ± 83.31	111.76 ± 98.42	0.26
Days in hospital	2.3 ± 1.2	2.9 ± 1.9	0.04
Screw diameter (mm)	7 ± 0.7	6.5 ± 0.3	< 0.001*
Screw length (mm)	47.7 ± 6.4	45.7 ± 4.3	< 0.001*
STIM threshold (mA)	34 ± 11.9	30.2 ± 90.8	0.002*

Data presented as mean ± standard deviation. Values were considered significant if *p* ≤ 0.05

*STIM* stimulation

There were 434 pedicle screws analyzed in the robotic group and 243 screws analyzed in the non-robotic group. The screw diameter and length were significantly greater in the robotic group compared to the non-robotic group (*p* < 0.001). Of the pedicle screws with STIM data available (619 screws), STIM threshold values for the robotic group were significantly higher than for the non-robotic group (*n* = 432; 34.0 ± 11.9 mA, and *n* = 187; 30.2 ± 9.8 mA, respectively; *p* = 0.002).

No complications occurred that were attributable to robotic technique. There were no implant failures and no revision or removal of implants within 1 year after the surgical dates for any patients. Within 1.5 years of the surgical dates, 1 patient in the non-robotic group suffered bilateral S1 pedicle screw fractures with pseudoarthrosis and required revision fusion surgery. No robotic surgery patients required hardware revision within 1.5 years of follow-up.

## Discussion

The present study compared pedicle screw sizes and placement safety using threshold stimulation measurements in patients who had surgery with or without robotic technology. Based on STIM values, it appears that robotic guidance allows for safe placement of pedicle screws as demonstrated by average STIM threshold. This held true even though, on average, 0.5 mm larger screws, which have the greatest risk of pedicle breach, were placed in the robotic group.

It is a common strategy to utilize pedicle screw fixation to stabilize the thoracic and lumbar spine in the instrumented correction of spinal pathologies [2, 22, 23]. Mispositioned pedicle screws may lead to a number of potentially life-threatening complications such as neurovascular damage, dural tearing, pain, and excessive bleeding, among others [24]. The free-hand technique with fluoroscopic guidance is currently the most utilized method of pedicle screw implantation. However, there are numerous disadvantages to this approach such as increased intraoperative radiation exposure and the potential of violation of facet joint during screw insertion (with less accurate screw positions) compared to robot-assisted surgery [24, 25].

Intraoperative validation of pedicle screw position is an extremely useful strategy for verifying screw position. One technique to check screw position is triggered EMG testing. Numerous studies have investigated the reliability of triggered EMG testing for identifying potential mispositioned screws, reporting a specificity of 0.94 [26]. Using triggered EMG testing provides the surgeon with real-time monitoring of potential hazards. Electrical stimulation near a nerve causes a subsequent muscle action potential from the myotomes innervated by the nerve roots close to the stimulated instrument [13]. Therefore, a positive response at a relatively low threshold is indicative of pedicle wall breach, and is considered a warning sign of poor implant position and potential neurologic injury [14, 15].

In recent years, robotic guidance systems have been developed as a means to decrease pedicle screw mispositioning. This is accomplished through the robot’s ability to use real-time image guidance and navigational capabilities [10, 20, 27–29]. Many studies claim that robotic guidance during surgery allows for improved screw placement with up to 99% screw placement accuracy, while others have reported equal or less accurate placement when comparing other methods of screw insertion with robotic technology [18–20, 30, 31]. These inconsistencies necessitate the need for studies such as this one to investigate the safety of using robotic technology in spinal surgery.

Results of the present study suggest that the use of robotic technology increases operative time by about 30 min. However, it should be noted that this study focused on the initial 100 consecutive patients after adoption of robotic technology. There is likely a learning curve associated with incorporating robotic technology, and operative times typically decrease with more experience and newer-generation software. For example, there was a significant difference (*p* < 0.01) in the average operative time in the robotic group between the first 14 cases of minimally invasive, one-level transforaminal fusion (TLIF) (177 min) and the last 14 cases (131 min). Additionally, by adopting the robotic technology, the surgeon could do more MIS surgeries so that a remarkable number of minimally invasive surgeries (one-third of the cohort) were performed in the robotic group, and that could have influenced the change in operative times. Patients who underwent surgery with the use of robotic technology had fewer postoperative days in the hospital compared to the non-robotic group. This finding is likely mostly due to the presence of minimally invasive cases in the robotic group, as minimally invasive approaches have been associated with shorter-length hospital stays [32]. While there was no significant difference in the length of hospital stays between robotic and non-robotic open cases (*p* = 0.18), the MIS robotic patients had significantly shorter lengths of stay compared to the open non-robotic cases (2.3 vs 2.9 days, respectively; *p* = 0.04).

This is the first study to use triggered EMG thresholds to compare pedicle screw placement in spinal surgery between robotic and non-robotic surgical patients. These thresholds can be seen as a surrogate marker of the safety of the implanted screw position. Based on the operative findings (Table 2), screw diameter and length were significantly greater in the robotic group. The use of larger screws is advantageous because it allows for increased stability [33, 34]. However, larger screw size can translate to increased risk of screw misplacement because there is less room for error [35]. Additionally, screws with wider diameters also have less electrical resistance, and this also could potentially lower the STIM threshold. Despite this, the robotic group still had significantly higher STIM values compared to the non-robotic group (*p* = 0.002), suggesting that surgeons may be able to use larger pedicle screws without compromising safety by utilizing robotic technology.

There are inconsistencies in the literature with regard to a STIM safety threshold [36, 37]. However, it does appear that most authors hover around 8 mA as a threshold to denote what is considered safe for the patient, with anything below 8 mA being potentially harmful, especially with the lumbar pedicle screws in which the test has a higher sensitivity and specificity [13, 37, 38]. Screw placement was acceptable and considered safe in both cohorts based on the average STIM thresholds (34 and 30 mA). However, since there were significantly higher STIM scores in the robotic group even though the screws were significantly larger, these results suggest that robotic assistance allows for larger screws to be placed without compromising patient safety.

These results should be interpreted within the confines of study limitations. The generalizability of these results is limited, as the current study is a retrospective review of data from a single surgeon at a single facility. These data suggest that the robotic technique may limit future problems and the need for additional surgery, since one patient in the non-robotic group required revision surgery within 1.5 years after the primary surgery, while no robotic surgery patients required revision within the same time frame. Future studies are needed, however, to determine conclusively if the larger pedicle screw sizes made possible with robotic technology actually translate into improved long-term clinical outcomes. The high STIM thresholds in this study suggest that the robotic technique allows for insertion of large screws without compromising safety.

## Conclusion

The results of the present study indicate that the use of robotic navigation and guidance during spinal surgery allows larger pedicle screws to be inserted without compromising safety, as determined by the surrogate marker of STIM threshold values.
